# Predicting Colorectal Cancer Recurrence and Patient Survival Using Supervised Machine Learning Approach: A South African Population-Based Study

**DOI:** 10.3389/fpubh.2021.694306

**Published:** 2021-07-07

**Authors:** Okechinyere J. Achilonu, June Fabian, Brendan Bebington, Elvira Singh, Gideon Nimako, M. J. C. Eijkemans, Eustasius Musenge

**Affiliations:** ^1^Division of Epidemiology and Biostatistics, School of Public Health, Faculty of Health Sciences, University of the Witwatersrand, Parktown, Johannesburg, South Africa; ^2^Medical Research Council/Wits University Rural Public Health and Health Transitions Research Unit (Agincourt), School of Public Health, Faculty of Health Sciences, University of Witwatersrand, Johannesburg, South Africa; ^3^Wits Donald Gordon Medical Centre, School of Clinical Medicine, Faculty of Health Sciences, University of Witwatersrand, Johannesburg, South Africa; ^4^Department of Surgery, Faculty of Health Science University of the Witwatersrand Faculty of Science, Parktown, Johannesburg, South Africa; ^5^National Cancer Registry, National Health Laboratory Service, 1 Modderfontein Road, Sandringham, Johannesburg, South Africa; ^6^Industrialization, Science, Technology and Innovation Hub, African Union Development Agency (AUDA-NEPAD), Johannesburg, South Africa; ^7^Julius Center for Health Sciences and Primary Care, University Medical Center, Utrecht University, Utrecht, Netherlands

**Keywords:** colorectal, cancer, recurrence, survival, machine learning, filter feature selection, prediction

## Abstract

**Background:** South Africa (SA) has the highest incidence of colorectal cancer (CRC) in Sub-Saharan Africa (SSA). However, there is limited research on CRC recurrence and survival in SA. CRC recurrence and overall survival are highly variable across studies. Accurate prediction of patients at risk can enhance clinical expectations and decisions within the South African CRC patients population. We explored the feasibility of integrating statistical and machine learning (ML) algorithms to achieve higher predictive performance and interpretability in findings.

**Methods:** We selected and compared six algorithms:- logistic regression (LR), naïve Bayes (NB), C5.0, random forest (RF), support vector machine (SVM) and artificial neural network (ANN). Commonly selected features based on OneR and information gain, within 10-fold cross-validation, were used for model development. The validity and stability of the predictive models were further assessed using simulated datasets.

**Results:** The six algorithms achieved high discriminative accuracies (AUC-ROC). ANN achieved the highest AUC-ROC for recurrence (87.0%) and survival (82.0%), and other models showed comparable performance with ANN. We observed no statistical difference in the performance of the models. Features including radiological stage and patient's age, histology, and race are risk factors of CRC recurrence and patient survival, respectively.

**Conclusions:** Based on other studies and what is known in the field, we have affirmed important predictive factors for recurrence and survival using rigorous procedures. Outcomes of this study can be generalised to CRC patient population elsewhere in SA and other SSA countries with similar patient profiles.

## 1. Introduction

Colorectal cancer (CRC) is the third most common cancer, and the fourth cause of cancer-related death (1). Approximately 2 million cases of CRC were diagnosed globally in 2018. The CRC incidence significantly varies, with high-income countries having a higher risk of CRC than low-middle-income countries (LMICs). However, this may not be the true reflection of the burden of cancer in LMICs due to the lack of cancer registries in most LMICs (2). CRC is steadily rising in LMICs because of the adoption of western lifestyle (3). South Africa (SA) has the highest incidence of CRC in sub-Saharan Africa, and CRC is among the most commonly diagnosed cancer in South African men, and women (4). In 2018, the age-standardised incidence rate of CRC in SA was 18.1 and 12.0 per 100,000 population of men and women, respectively (3).

Accurate prognosis of cancer outcomes can provide helpful knowledge to clinicians, which is critical in making informed decisions that can improve patient care. Several efforts have been invested in improving the accuracy of cancer outcome predictions both at the data level, and algorithmic level (5). The advancement in the amount of medical data generated in cancer research has enabled the development of various artificial intelligence and machine learning (ML) expert systems. In data mining (DM), these systems are used to identify risk factors that can support medical decisions in cancer prognosis. ML algorithms are built upon the foundation of statistical learning, but with fewer assumptions (6). Unlike statistical models, ML algorithms avoid the hurdles in accurately modelling the data-generating process and estimating the feature coefficients. Also, ML models are designed to automatically handle noise in a dataset, complex interaction, non-linearity, large sample size and features. Overall, ML algorithms have been shown to improve treatment outcome in cancer research (5, 7). However, ML only focuses on optimising the predictive performance rather than transparency and interpretability (7). Hence, ML and statistical models can be used concurrently to achieve both clarity and higher predictive power.

Several predictive models have been published in the area of CRC recurrence and survivability prediction using the concept of statistical and ML algorithms. Nan et al. (8) conducted a retrospective study on elderly patients with CRC, using a Cox proportional hazard model. These patients were followed for more than 5 years, and different optimal treatment methods were given to them. Their findings suggest that patient features such as age, treatment methods, lymph node metastasis, histology type, Dukes stage and degree of differentiation should be considered when planning a patient's treatment method. Using a support vector machine (SVM) algorithm, Ting et al. (9) highlighted that features including age, tumour size, pathologic stage, smoking, alcohol consumption, organisational patterns, BMI are important predictive factors for CRC recurrence. In the prediction of a 5-year CRC patient-specific survival outcome, Bychkov et al. (10) developed a deep learning-based classifier directly from small digitised tumour tissue samples. Although with an area under the curve of receiving operating classification (AUC) of 69%, the deep learning classification outperformed the predictive classification accuracy achieved by a pathologist assessment, with more prognostic information. Previous studies have shown that factors affecting CRC recurrence and patients survival with CRC are not fully understood. These factors vary across studies due to differences in geographical locations, lifestyle and available patient records.

Overall, several clinical information, modelling strategies, and algorithms have been employed in the CRC predictive studies (9–13). Algorithms including SVM, artificial neural network (ANN), random forest (RF), C5.0, Naïve Bayes (NB) and logistic regression (LR) have shown good performance in predicting survivability and recurrence of CRC (11, 13, 14). Nonetheless, there is no optimal model in the literature, and most of the developed models are yet to be validated. Hence, there exists a necessity to develop a South African model that can identify risk factors influencing CRC recurrence or survival and serves as a helping hand for specialists in personalising patients' medical regimen. This study predicted CRC recurrence and survivability outcomes using C5.0, LR, RF, NB, SVM, and ANN. Our aims were to (i) quantify and compare the performance of these algorithms using both established and local information on the CRC patient database, (ii) identify features that have predictive value on CRC patient recurrence and survival, and (iii) evaluate the reliability and stability of the model performance using different simulated data. Thus, this study will form the basis of other studies in developing a South African model for CRC prognostic prediction.

## 2. Materials and Methods

### 2.1. Study Population

The Colorectal Cancer in South Africa (CRCSA) study was the first prospective study designed to describe the clinical presentation, demographics, risk factors, treatment, and outcomes according to population group, from both private and state health–care facilities in Johannesburg, SA (2). This study was officially launched in January 2016. This study aimed to describe the clinical features and outcomes of adults presenting with histologically confirmed primary adenocarcinoma of the colon and rectum in a multi-ethnic urban population in Johannesburg. Participants over the age of 18 years with CRC within the previous 12 months were eligible. A total number of 716 patients were recruited from 2015 and were followed up at 6 months intervals from the date of recruitment, with the last follow up to the 31st of March 2020. Charlotte Maxeke Johannesburg Academic Hospital (CMJAH), Chris Hani Baragwanath Academic Hospital (CHBAH), Wits Donald Gordon Medical Centre (WDGMC), and Edenvale Hospital that serve as private and public hospitals to many urban dwellers in the Johannesburg metropole were used as the study sites. However, the database for the CRC study was curated at WDGMC; hence, we referred to this study data as WDGMC CRC data. Questionnaires and scoring systems were used for the baseline assessments at these sites. Patient information included demographics, socio-economic status, dietary history, family history of cancer, medical and surgical history, colonoscopy, histopathological diagnosis, recurrence and survival histories. The design and methodology of the study data are detailed in Bebington et al. (2). In collaboration with authors in Bebington et al. (2), the present study was approved by the Human Research Ethics Committee (Medical) of the University of the Witwatersrand (M1911131).

### 2.2. Predictive Model Development and Validation

Basic descriptive statistics were performed to describe the characteristics of the WDGMC CRC data for the continuous and categorical features in the dataset. Eighty-eight (88) features were identified in the CRC dataset based on previous studies and expert opinion. Several data pre-processing steps were performed to reduce algorithm-deployment time and improve the quality and efficiency of our findings. The pattern of missing values within the features was assessed, and Supplementary Figure 1 shows that the feature “reason for the use of chemotherapy” has the highest proportion of missing values. The Little MCAR test showed that the missingness in the data was completely random (*p* = 0.306). MissForest method of imputation (15) was used to impute the missing values in the WDGMC CRC data. This method of imputation has successfully been applied in different fields of research, including our recent study (16), with minimum error in both the continuous and categorical features. A total number of 696 patients were selected for analysis after data pre-processing. Two variations of the WDGMC data were used in this study to model recurrence and survivability. The first outcome feature (recurrence) is a binary categorical variable, where the class values indicate disease recurrence (264 cases) or non-recurrence (433 controls), irrespective of whether it is a local or distant recurrence. The second outcome feature (survivability) is also a binary categorical variable with values “survived” (399 controls) or “not-survived” (281 cases). Sixteen patients that were censored during the follow-up were further dropped in the survival modelling because their outcome status is unknown.

Some of the identified features may not have an impact on the outcome variables in this study. Also, we understand that over-fitting a predictive model may affect its ability to generalise in other settings. To reduce the chances of over-fitting the classifiers, we considered reducing the number of study features. A systematic combination of univariate (Information gain and One Rule) and multivariate (Least Absolute Shrinkage and Selection Operator) feature selection methods were applied in this study. Information gain (IG) and One Rule (OneR) are filtering approaches with good performance reported in different practice settings (17, 18). These algorithms assign a score to each feature to indicate its impact on the outcome. The Least Absolute Shrinkage and Selection Operator (LASSO) fit a model containing all the study features and uses a regularisation process to penalise the coefficients of the predictors, thereby shrinking features that have no impact on the outcome to zero (19).

**Figure 1:**
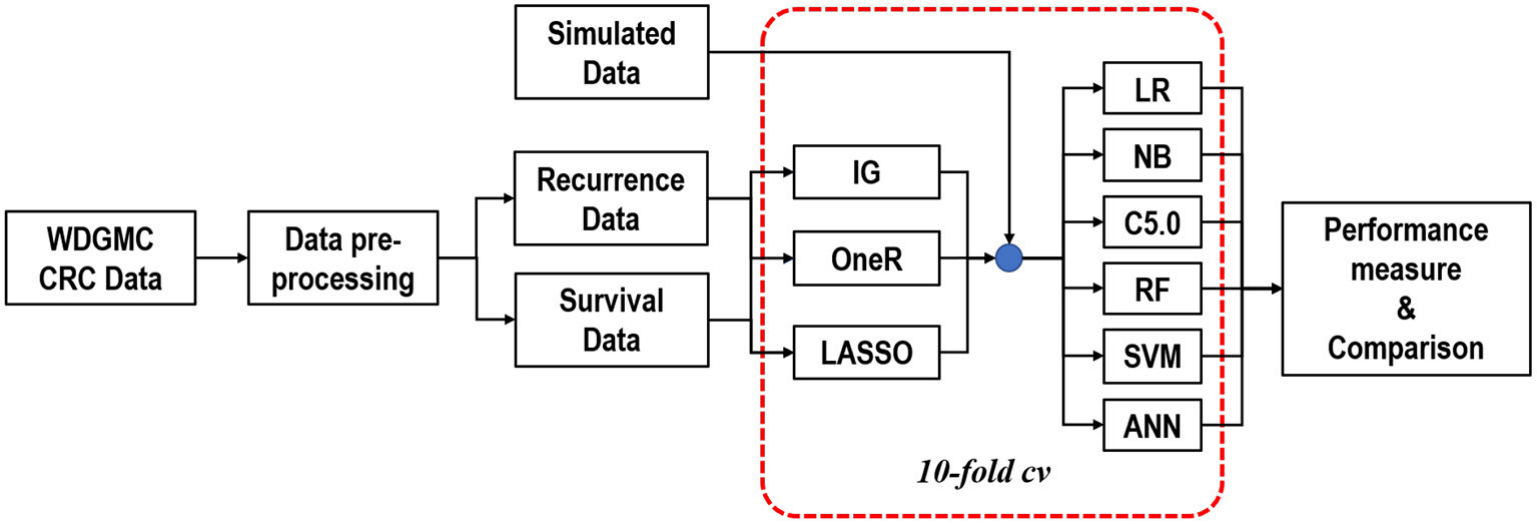
Graphical representation of the modelling approach to predicting CRC recurrence and survival.

A two-level feature selection method was proposed. We aimed to select and rank the top ten features for the univariate selection methods in the first level. For the LASSO method, we identified features with non-zero coefficients after the shrinking process. Common features selected in the first level were combined in the second level selection and were used as input features for the classifiers. It should be noted that this step was internally conducted within 10-fold cross-validation (CV) to give all the predictor features a fair advantage of being selected (Figure 1). In this procedure, the algorithms iterate 10-times over the study dataset. In each round, the dataset was split into 10-folds: 1-fold for validation and the remaining *k*−1 folds (nine folds) for training the model. The training-folds were used for the model establishment, while the testing fold was used to test the generalisability of the model.

We used LR, NB, C5.0, RF, SVM, and ANN algorithms for predictive analytics (20–23). For all the models implemented in this study, SVM gave the highest execution time of 29.02 s. Simulation studies were used to assess the stability and validity of the developed models (24). The simulation scenarios aimed to mimic the distribution of the WDGMC CRC data, based on the features used to develop the predictive models for recurrence and survival. The area under the curve of receiving operating characteristics (AU-ROC) was used to assess the discriminatory power of the predictive models, which was supported with a threshold (accuracy) metric (25). To evaluate the predictive performance of the models, the Wilcoxon signed-rank test was used to assess the performance differences among the classifiers based on the AU-ROC scores (26). A comprehensive description of the simulation methodologies, the selected algorithms and the parameters used in the model development is in the (Supplementary 1, 2 and Supplementary Table 2). We have also documented and shared the R codes used for all the analysis reported in this manuscript through Github (https://github.com/KechJay/ML_RSPM) for model reproducibility. However, only the synthetic data is included in this link, and the WDGMC data will be made available on request.

## 3. Results

### 3.1. Patient Characteristics and Feature Selection

The median length of follow-up for survival was approximately 11 months. Analysis of the demographics characteristics shows that the study was a predominance of black patients (356, 51%). Of this number, 154 (43%) experienced CRC recurrence, while 185 (52%) patients died. Among the 246 (35%) white patients, there were 78 (32%) recorded recurrence and 68 (28%) deaths. The remaining 95 (14%) patients (grouped as “others”) include Asians and mixed race. The records show that 32 (34%) of these group had recurred CRC, while 28 (29%) of the total died before the end of the study. It was observed that 44% of the male patients (364) died and 39% had recurrent cancer. Approximately 37% of the percentage of female patients (333) died or had CRC recurrence. The average age of the participants was 57 years, with a range of 18–91 years. Other features used in the modelling procedures are also described in Table 1.

**Table 1:** Characteristics of the WDGMC population based on the selected features from information gain, OneR, and LASSO.

**Feature**	**Total (%)**	**Description**	**Recurrence outcome**			**Survival outcome**		
			**IG**	**OneR**	**LASSO**	**IG**	**OneR**	**LASSO**
**Age at 1st visit**	57 (13)	Age at the time of first visit	0.022	0.621	–0.010			
**Race**		Race				0.042	0.567	0.009
Black	356 (51.1)							
White	246 (35.3)							
Others	95 (13.6)							
**Histology**		Histology				0.127	0.755	–2.527
Adenocarcinoma	430 (61.7)							
Others	267 (38.6)							
**CRC complications**		cancer related complication	0.036			0.098	0.708	–0.732
No	310 (44.5)							
Yes	387 (55.5)							
**Procedure**		Did patient undergo any procedure	0.031	0.641	–0.006	0.087	0.715	0.232
Yes	410 (58.8)	listed						
No	287 (41.2)							
**Hospital**		Study site of recruitment	0.018	0.621	-0.111	0.075	0.659	0.284
Private	248 (35.6)							
Public	449 (66.4)							
**Language**		What is your home language				0.060	0.645	
English	241 (34.6)							0.000
Indigenous African language	326 (46.8)							0.680
Others	130 (18.7)							1.316
**Radiological stage**		Assessment of stage of	0.141	0.781		0.062	0.673	
Unable to stage	80 (11.5)	malignancy			0.000			0.794
Stage I and II	157 (22.5)				0.000			0.000
Stage III	240 (34.4)				-0.023			0.783
Stage IV	220 (31.6)				2.055			1.472
**Recurrence status**		Did patient cancer recur after				0.037	0.648	1.700
Recurrence	433 (62.1)	the follow-up						
Non-recurrence	264 (37.9)							
**Chemotherapy**		Receipt of chemotherapy	0.051	0.683	0.884			
Yes	246 (35.3)							
No	451 (64.7)							
**Treatment decision**		Treatment decision, MDT1	0.049	0.686	0.475			
Chemotherapy	214 (30.7)							
No chemotherapy	483 (69.3)							
**Prior CRC treatment**		What previous treatment was	0.028	0.666	1.205			
Surgical	68 (10.0)	given for this of patients colorectal						
Non-surgical	629 (90.0)	cancer prioir to recruitment						
**CRC prior to recruit**		Was this colorectal cancer	0.018	0.651	0.594			
Yes	112 (16.1)	diagnosed prior to recruitment						
No	585 (83.9)							
**Prior colonoscopy**		Colonoscopy done prior	0.023	0.634	0.157			
Yes	451 (64.7)	to first visit to the colorectal						
No	246 (35.3)	unit						

Table 1 also shows the best combination of features based on the three methods of feature selection for recurrence and survival outcomes. Under the univariate techniques, the ranking scores of these features show the importance of each feature to the outcome. For instance, the two methods indicate that having adenocarcinoma or non-adenocarcinoma (based on histology report) is the most relevant feature for survival. This feature was not selected by any of these methods under recurrence. The most important feature selected by these methods under recurrence is the stage of CRC malignancy. CRC staging was also selected by these methods as an important feature for recurrence. The LASSO method shows the coefficients of the selected features, indicating their effects on the outcomes at λ = 0.032, where λ is the tuning parameter that controls the degree of penalty.

**Figure 2:**
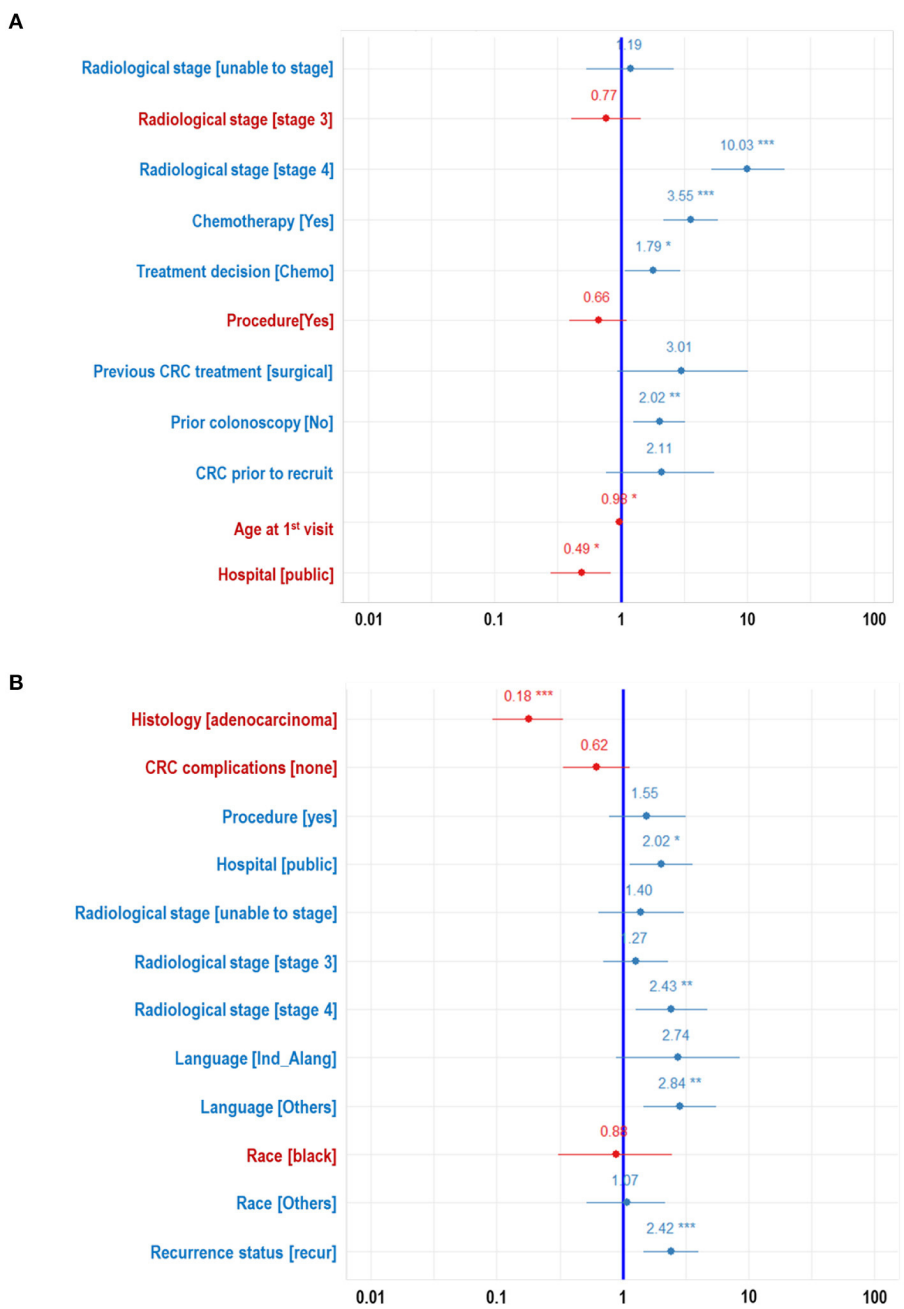
Forest plots developed from logistic regression showing the effects of each features on the WDGMC CRC **(A)** recurrence and **(B)** survival. Features with significance effects are shown with asterisks. Features with their effects values written in red letters decrease odds of CRC recurrence or CRC survival.

The ranking of the importance of the selected features according to the different predictive models is shown in Tables 2, 3. Although all the features have contributed to the model developments, there is no consistency in the feature ranking across the predictive models. For the CRC recurrence modelling, the top-ranked features based on the models include “radiological stage”, “age at 1st visit”, “chemotherapy” and “hospital”. Also, “histology”, “radiological stage”, “CRC complication” is among the most important features for CRC survival (Table 3). We used LR to estimate the effects of these features on the outcome variables. The odds ratios are shown in Figures 2A,B for recurrence and survival, respectively. The figures show that some of these features negatively influence CRC recurrence or survival. For instance, we observed that the odds of CRC recurrence are reduced by 51% for those patients recruited at the public hospitals compared with patients recruited at the private hospital. The radiological stage, among other features, significantly increases the odds of CRC recurrence and survival. It is shown that the odds of CRC recurrence at stage IV is about 10 times higher than the odds when CRC is at stages I and II. We also observed that the odds of death at stage IV is more than twice the odds of death at stages I and II. Nevertheless, some of these important features used for the development of CRC recurrence or survival model show no significant effect on the outcomes (at 5% level of significance) according to LR.

**Table 2:** Risk factor ranking in descending order showing the relative importance of each feature to modelling WDGMC CRC recurrence as ranked by each predictive models.

**Rank**	**LR**	**NB**	**C5.0**	**RF**	**SVM**	**ANN**
1	Radiologic stage	Radiologic stage	Prior colonoscopy	Radiologic stage	Radiologic stage	Age at 1st visit
2	Chemotherapy	Chemotherapy	Radiologic stage	Age at 1st visit	Chemotherapy	Chemotherapy
3	Hospital	Treatment decision	Age at 1st visit	Chemotherapy	Treatment decision	Radiologic stage
4	Treatment decision	Procedure	Chemotherapy	Treatment decision	Procedure	Procedure
5	Age at 1st visit	Age at 1st visit	Treatment decision	Hospital	Age at 1st visit	CRC prior to recruit
6	Prior colonoscopy	Prior colonoscopy	Procedure	Procedure	Prior colonoscopy	Prior colonoscopy
7	Procedure	CRC prior to recruit	Prior CRC treatment	Prior CRC treatment	CRC prior to recruit	Treatment decision
8	Prior CRC treatment	Prior CRC treatment	Hospital	Prior colonoscopy	Prior CRC treatment	Hospital
9	CRC prior to recruit	Hospital	CRC prior to recruit	CRC prior to recruit	Hospital	Prior CRC treatment

**Table 3:** Risk factor ranking in descending order showing the relative importance of each feature to modelling WDGMC CRC survival as ranked by each predictive models.

**Rank**	**LR**	**NB**	**C5.0**	**RF**	**SVM**	**ANN**
1	Histology	Histology	Histology	Histology	Histology	CRC complications
2	Recurrence status	CRC complications	Hospital	Hospital	CRC complications	Radiological stage
3	Hospital	Procedure	Radiological stage	Radiological stage	Procedure	Histology
4	Radiological stage	Hospital	Recurrence status	CRC complications	Hospital	Hospital
5	Language	Radiological stage	Language	Procedure	Radiological stage	Recurrence status
6	CRC complications	Language	CRC complications	Recurrence status	Language	Race
7	Procedure	Recurrence status	Race	Language	Recurrence status	Procedure
8	Race	Race	Procedure	Race	Race	Language

### 3.2. Model Predictive Performances for Recurrence and Survival Outcomes

Table 4 shows that all the resulting models had AU-ROC above 0.85. ANN demonstrated the highest performance with discriminating scores of 0.87 (CI: 0.835–0.905). It should be noted that the confidence interval of the models do not include the 50% chance (*y* = *x*), indicating that these models are significantly better than chance. The least predictive performance for the CRC recurrence was achieved by NB (0.854, CI: 0.819–0.890). Even though the ANN had the highest AU-ROC value, pairwise comparisons of the model performances show no significant difference between ANN and the other models. The results of the stability assessment using simulated data are also shown in Table 4. All the AU-ROC scores for the different predictive models achieved more than 90% discriminative accuracy in predicting CRC recurrence. ANN maintained higher predictive performance across the three simulated datasets. In the same manner, the ANN has the highest AU-ROC value in predicting survival (0.818, CI: 0.781–0.856) as shown in Table 5. Other predictive models achieved AU-ROC curves higher than 80%, with RF and SVM showing the least performance scores. There was no significant difference when these models were compared. It is observed that all the predictive models across the simulated data show comparable estimates. Furthermore, the models show comparable performance in predicting recurrence and survival, based on the accuracy metric (Supplementary Table 3).

**Table 4:** AU-ROC performance scores (with confidence interval) examining the consistency of the predictive models from the WDGMC CRC recurrence data and across the three simulated datasets used for model validation.

**Model**	**WDGMC (*N* = 697)**	**Sim_Data (*N* = 697)**	**Sim_Data (*N* = 3,485)**	**Sim_Data (*N* = 6,970)**
	**AUC (95% CI)**	**AUC (95% CI)**	**AUC (95% CI)**	**AUC (95% CI)**
LR	0.861 (0.840–0.899)	0.941 (0.919–0.964)	0.923 (0.917–0.930)	0.927 (0.922–0.932)
NB	0.854 (0.819–0.890)	0.932 (0.908–0.965)	0.925 (0.917–0.933)	0.925 (0.921–0.929)
C5.0	0.867 (0.831–0.903)	0.929 (0.904–0.954)	0.937 (0.931–0.943)	0.945 (0.943–0.948)
RF	0.863 (0.828–0.898)	0.931 (0.905–0.957)	0.933 (0.925–0.941)	0.945 (0.941–0.949)
SVM	0.867 (0.833–0.900)	0.940 (0.918–0.963)	0.923 (0.916–0.930)	0.930 (0.907–0.963)
ANN	0.870 (0.835-0.905)	0.955 (0.940–0.971)	0.947 (0.942–0.951)	0.953 (0.949–0.958)

**Table 5:** AU-ROC performance scores (with confidence interval) examining the consistency of the predictive models from the WDGMC CRC survival data and across the three simulated datasets used for model validation.

**Model**	**WDGMC (*N* = 680)**	**Sim_Data (*N* = 680)**	**Sim_Data (*N* = 3,400)**	**Sim_Data (*N* = 6,800)**
	**AUC (95% CI)**	**AUC (95% CI)**	**AUC (95% CI)**	**AUC (95% CI)**
LR	0.816 (0.776–0.856)	0.912 (0.893–0.930)	0.907 (0.897–0.916)	0.911 (0.905–0.918)
NB	0.811 (0.771–0.850)	0.907 (0.891–0.923)	0.904 (0.893–0.914)	0.907 (0.901–0.914)
C5.0	0.811 (0.771–0.855)	0.902 (0.886–0.917)	0.906 (0.897–0.914)	0.911 (0.904–0.918)
RF	0.806 (0.769–0.843)	0.893 (0.876–0.909)	0.900 (0.890–0.910)	0.907 (0.900–0.914)
SVM	0.806 (0.734–0.847)	0.910 (0.893–0.927)	0.907 (0.897–0.916)	0.911 (0.904–0.917)
ANN	0.818 (0.781–0.856)	0.911 (0.893–0.929)	0.909 (0.900–0.918)	0.913 (0.907–0.920)

## 4. Discussion

In this study strategy, we imputed a few cells with missing information in the data to maximise the cases in the analysis. The idea of selecting the best common features based on the systematic combination of univariate and multivariate feature selection methods optimised the accuracy of the prediction models. This was done to minimise the chances of over-fitting by not crowding the algorithms with too many features (27). Reducing the cost of measuring several features for a specific outcome, mainly when fewer features can be used to represent others, is the goal of prediction modelling. When we incorporated the features that were not commonly identified by the feature selection method, the models' performances decreased compared to when the commonly identified features were used. This underscores the significance of systematically employing more than one feature selection method in this type of study. Our findings agree with Hastie et al. (28) that feature selection before cross-validation may result in a selection bias that could impact the predictive performance of a model. We observed differences in selection and performance before and within the 10-fold CV, which supported the study by Hastie et al. (28). A 10-fold CV was employed to improve the reliability of the resultant estimates from the classifiers. This CV method was considered because the sample size is relatively small, and this method has been reported to provide the best trade-off between bias and variance in a relatively small dataset (29).

We found that these models demonstrated a high and comparable predictive ability (based on the AU-ROC) and showed no significant difference in their performance. Our findings recommend that all these models should be considered in modelling CRC recurrence and survival. The two statistical methods (NB and LR) were as robust as ML predictive models, irrespective of the complexity of the ML algorithms. Our study correlates with other studies that found no significant difference between LR and ANN or SVM (30, 31). Studies on CRC recurrence and survival have used different prediction strategies such as modelling only rectal or colon, individual stage of CRC, year of survival, different features and modelling procedures. Our study used data from all stages of CRC, incorporating all the survival years in the data. Performance may not be directly compared; however, our predictive models discriminated reasonably well, both in recurrence and survival, and achieved AU-ROC values comparable to other CRC studies (9–13).

The concept of model validation through data simulation shows that the algorithms could identify the signals correlated with the outcome features. The simulated data over-simplified the artificial signal, which improves the models' performance compared to the real (WDGMC) datasets. Moreover, the stability and validity of the model predictions across the different simulated samples provided empirical evidence that supports the results of the real data. Besides, when the artificial signals were removed from the simulated data, the discriminative ability of the algorithms deteriorated because patient risk (of “survived” or “not survived”) became less separable. This supports the evidence that predictive models can be compromised negatively in the absence of signal in a dataset (24).

We revealed several local and established risk factors for CRC recurrence and survival. Most of the factors, including CRC grading, gender, marital status and education level, etc., significantly influence the outcomes univariately. However, we aim to develop our model with a set of features (risk factors) that could significantly influence the outcomes. Hence, only features that were commonly selected in the modelling procedures were incorporated into the predictive models. Recurrence is a well-known risk factor that influences the survival of CRC patients (32). Our study shows that patients who experienced recurrence had poorer survival than patients who did not have a recurrence. “Radiological stage” is an important feature in this study, which significantly influences recurrence and survival. Specifically, “stage IV” CRC (when compared with the combination of “stages I” and “II”) increases the chances of recurrence or death. Studies, including Nan et al. (8), reported stage IV CRC patients to have a poor prognosis. A previous study reported that younger patients (<50 years) with CRC experienced higher recurrence compared to older patients but have a comparable rate of survival (33). This corresponds with our study, which shows that the odds of recurrence slightly decreases with an increase in age. The age-related disparity is a well-documented fact and has been linked to factors such as a late-stage diagnosis and lifestyle (33, 34).

The type of CRC cancer is another important risk factor that significantly influences the survival outcome. The importance of this factor was affirmed in previous studies including Nan et al. (8) and Stojadinovic et al. (35). Our study indicates that patients with adenocarcinoma-type CRC had a reduced survival rate compared with patients presented with non-adenocarcinoma CRC. The impact of the recruitment site on recurrence or survival has been noted in the result section. A recent study in SA showed improved survival for CRC patients treated in private hospitals (36). However, this study was not extrapolated to CRC patients that were managed in public hospitals. Our findings support an improved survival for patients treated in a private hospital compared to those treated in public hospitals. These observed survival differences could be linked to the fact that most patients in public hospitals are relatively poor with lower education level, have difficulties accessing medical care and tend to have more advanced disease stage at the time of presentation. All these factors are likely to impact their survival outcome. Concerning recurrence, one would expect patients in a private hospital to have lower odds of recurrence than patients in a public hospital. However, the patients in public hospitals showed lower odds of CRC recurrence. The only plausible explanation is that patients in private hospitals survive longer and experience recurrence than patients in public hospitals. This compliments our observed difference in survival between the private and public sectors because recurrence can only occur in patients that are alive.

Several studies [as discussed in a review by Wolpin and Mayer (37)] identified the benefit of treatment with chemotherapy (alone and /or in combination with surgery or radiation) to reduce the risk of CRC recurrence and mortality in some patients with specific risk groups. In our study, we pooled all the treatments with chemotherapy to create a new variable called “treatment with chemotherapy, yes/no”, irrespective of receiving any other treatment options. Besides, our predictive models were developed for all stages of CRC, with higher frequencies (65%) of the patients diagnosed with stages III and IV CRC. This study showed that intervention with chemotherapy was not helpful as the predictive model did not select this intervention as a factor indicating a favourable prognosis. This is reasonable, as such modalities are only employed in patients with an advanced CRC stage, which drives the poor outcomes. On the other hand, recurrence of CRC could be associated with several risk factors, including molecular sub-typing, stage of CRC, the primary site of CRC and treatment of co-morbidities (37, 38).

## 5. Conclusion

This study has certain limitations and strengths. We dropped 17 (2.4%) cases that were lost to follow-up in the survival models to avoid introducing bias to the estimations. Nonetheless, 71% of these cases were patients from public hospitals; hence, the exclusion of these patients should not alter the supposition that the private hospital had higher survival than public hospitals. In a future study using this data, we will predict the survival trend of these patients while incorporating censored cases in the analysis. Also, a future study using this data could explore the capabilities of other models not included in this study and disintegrate the CRC stages to uncover more trends within this population. This study strongly suggests that statistical algorithms should be concurrently used with ML algorithms to enhance global interpretation. We recommend that physicians should consider the important features noted in our findings when selecting promising therapeutic strategies. This type of study could stimulate the extrapolation of data collection outside of the hospitals because the variables that determine the outcome are as much to do with the pre-hospital detection of CRC patients as it is the treatment given to them by clinicians. We believe that this will be important for clinicians to grasp and engage in this type of study to embrace the precepts of primary health. Besides, the findings of this study will be beneficial to CRC researchers in other parts of the country. SA needs this level of data interpretation, especially in a circumstance where there is diversity and inequality in the country's demographic landscape.

In conclusion, we have analysed a CRC study covering patients who visit both the private and public hospitals in the most populous city in SA. This study developed and internally validated the recurrence and survival prediction models for South African CRC patients. External validation of these models could have further affirmed the validity of this study. However, there is an ongoing framework for a prospective study to collect data for future validation of this study. The findings of this study form the basis for further studies on CRC in SA, using ML approaches. Also, this study can be generalised, not only to the population of CRC patients in SA but in other SSA countries with similar trends in urbanisation and dynamics in CRC epidemiology.

## Data Availability Statement

The raw data supporting the conclusions of this article will be made available by the authors, without undue reservation.

## Ethics Statement

The studies involving human participants were reviewed and approved by Human Research Ethics Committee (Medical) of the University of the Witwatersrand (M1911131). The patients/participants provided their written informed consent to participate in this study.

## Author Contributions

OA conceptualised, analysed the data, compiled the results, and wrote the manuscript. BB and JF provided the data and reviewed the manuscript. ES and GN reviewed the manuscript. MJCE and EM supervised the concept development and reviewed the manuscript. All authors contributed to the article and approved the submitted version.

## Acknowledgements

We want to acknowledge the patients that participated in the CRCSA study and the staff of WDGMC that curated the database, especially Mrs Soneni Maphosa, for her valuable explanation of the study design and variables.

## Conflict of Interest

The authors declare that the research was conducted in the absence of any commercial or financial relationships that could be construed as a potential conflict of interest.
