# Corrigendum: Predicting Colorectal Cancer Recurrence and Patient Survival Using Supervised Machine Learning Approach: A South African Population-Based Study

**DOI:** 10.3389/fpubh.2021.778749

**Published:** 2021-10-29

**Authors:** Okechinyere J. Achilonu, June Fabian, Brendan Bebington, Elvira Singh, Gideon Nimako, M. J. C. Eijkemans, Eustasius Musenge

**Affiliations:** ^1^Division of Epidemiology and Biostatistics, School of Public Health, Faculty of Health Sciences, University of the Witwatersrand, Parktown, Johannesburg, South Africa; ^2^Medical Research Council/Wits University Rural Public Health and Health Transitions Research Unit (Agincourt), School of Public Health, Faculty of Health Sciences, University of Witwatersrand, Johannesburg, South Africa; ^3^Wits Donald Gordon Medical Centre, School of Clinical Medicine, Faculty of Health Sciences, University of Witwatersrand, Johannesburg, South Africa; ^4^Department of Surgery, Faculty of Health Science University of the Witwatersrand Faculty of Science, Parktown, Johannesburg, South Africa; ^5^National Cancer Registry, National Health Laboratory Service, 1 Modderfontein Road, Sandringham, Johannesburg, South Africa; ^6^Industrialization, Science, Technology and Innovation Hub, African Union Development Agency (AUDA-NEPAD), Johannesburg, South Africa; ^7^Julius Center for Health Sciences and Primary Care, University Medical Center, Utrecht University, Utrecht, Netherlands

**Keywords:** colorectal, cancer, recurrence, survival, machine learning, filter feature selection, prediction

In the published article, **Gideon Nimako** was not included as an author in the published article. The authors apologize for this error and state that this does not change the scientific conclusions of the article in any way. The original article has been updated.

## Publisher's Note

All claims expressed in this article are solely those of the authors and do not necessarily represent those of their affiliated organizations, or those of the publisher, the editors and the reviewers. Any product that may be evaluated in this article, or claim that may be made by its manufacturer, is not guaranteed or endorsed by the publisher.

