# Supplementary material for: Predicting Colorectal Cancer Recurrence and Patient Survival Using Supervised Machine Learning Approach: A South African Population-Based Study
Source: Front Public Health. 2021 Jul 7;9:694306. doi: 10.3389/fpubh.2021.694306 (PMC8292767; doi:10.3389/fpubh.2021.694306)
Supplement: Supplementary file 1 [file Data_Sheet_1.PDF]

Predictive Models for Colorectal Cancer Recurrence and Patient Survival: A Johannesburg Study, South Africa

Supplementary Section

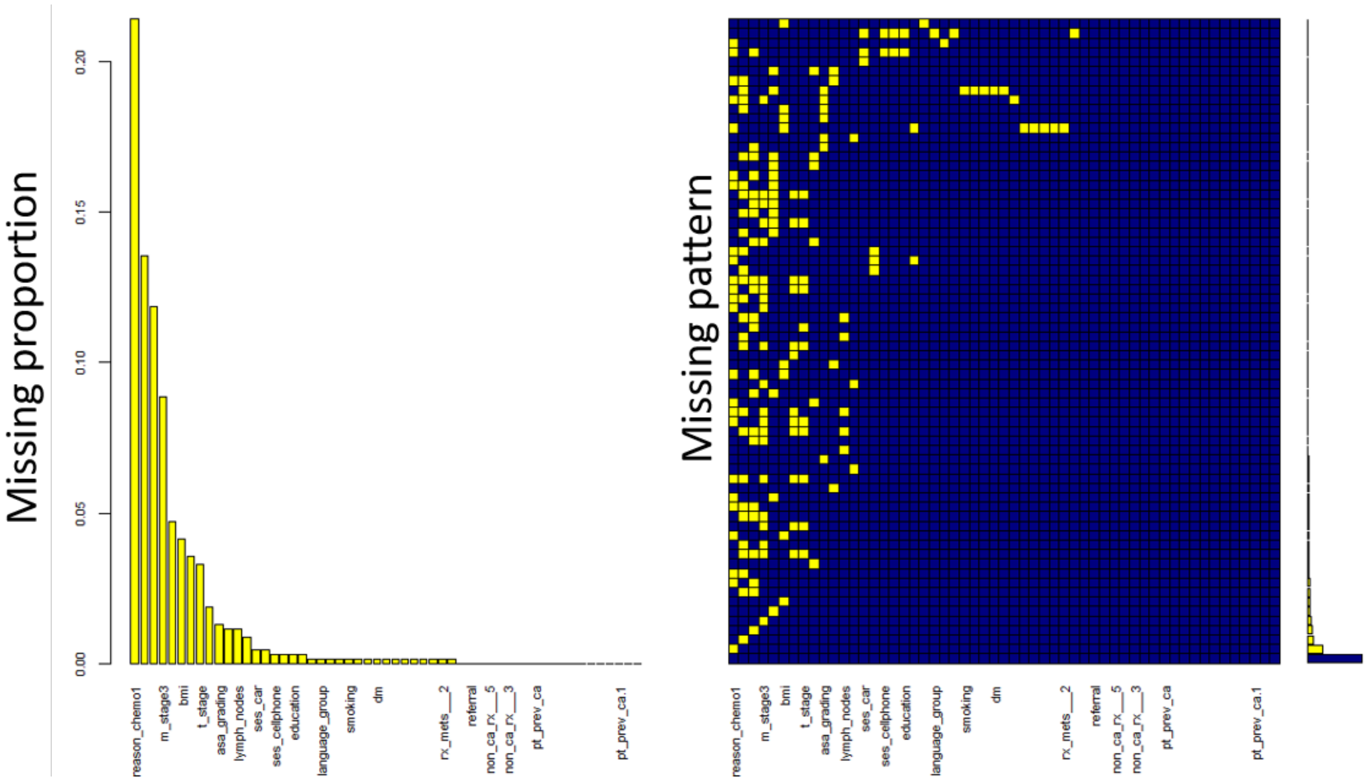

Figure 1: Pattern and proportion of missingness in the CRCSA study.

1 Simulated datasets

Simulation studies were used to evaluate the stability and validity of the predictive models. The simulation scenarios aimed to mimic the distribution of the WDGMC CRC data, based on the features used to develop the predictive models for recurrence and survival. At first, we simulated data equal to the sample sizes used in the model development for the two outcomes. To explicitly validate consistency in performance, we varied the sample sizes by multiplying each of the study samples by a factor of 5 and 10 with the notion that increasing the sample size may not affect the stability of the model performance. The relative risk or effect size (of each feature) used for the data simulation were average effects of 10000 bootstrap samples on each variation of the WDGMC data. A baseline probability was randomly assigned to each individual, while linearly aggregating the effect sizes with the simulated features. To generate the outcome variable, the aggregated features were added to the baseline probability and a cut-off mark of the final probability was chosen to classify each simulated case as “survived” or “not survived”/“recur” or “non-recur”. The distribution of the generated features was compared with the WDGMC data distribution to ascertain the results of the simulation.

2 Choice of classifier

Given the CRC training data $S = \{x_i, y_i\}$, for $i = 1, 2, \ldots, n$, where $x_i \in \mathbf{R}^d$ is the CRC patient feature vector and $y_i \in \{-1, +1\}$ is the target outcome (i.e recurrence and non-recurrence), we have provided a brief review of the properties of the selected classifiers as highlighted below:

2.1 Logistics regression

Binary logistic regression is a statistical model used for classification problems when the response variable is dichotomous [1]. The binomial distribution posses odds ratio (OR), where the log odds of the outcome is modelled as a linear combination of the features x_i. The method of maximum likelihood is used to estimate the coefficients (w_0, w_i) of the features. The intuition behind using maximum likelihood is to find the estimates of w_0 and w_i that can predict the probability of survival i.e. close to zero if a patient did not survive beyond 3 years, and 1 if a patient survived beyond 3 years. The probability $P(y = 1|x)$ is estimated as follows

$$P(y = 1|x_i) = \frac{1}{1 + e^{-(w_0 + w_1 x_1 + \cdots + w_n x_n)}} \quad , \tag{1}$$

where w_0 is the intercept, $w_i(i = 1, 2, \ldots, n)$ is the parameter vector and $x_i(i = 1, 2, \ldots, n)$ is the feature vector. LR has become a well known standard tool in the data mining (DM) field and can result in a comparable performance with more recent classifiers in some settings. In addition, the resulting output is more transparent and interpretable than the recent classifiers.

2.2 Naïve Bayes

Naïve Bayes (NB) is a simple probabilistic classifier developed from Bayes theorem. This theorem is very useful but makes a strong assumption on the independence between the features given the class [2]. In other words, it assumes the individual features contribute equally and independently towards the final decision. This indicates the NB algorithm simplifies a multivariate problem to a univariate problem since dependencies such as correlation among the input features are ignored. Although the assumption of independence may appear unrealistic in real-world practice, the NB classifier has shown a comparable performance with more sophisticated algorithms. The class conditional probability states:

$$P(y|x_i) = \frac{P(y)P(x_i|y)}{P(x_i)} \tag{2}$$

For any new case, the class is determined by computing and comparing the estimated conditional probabilities of the outcome class $y_i(-1, 1)$. The higher the estimated value for any of the classes, the more likely the new case belongs to that class.

2.3 Decision tree

Decision trees (DT) are transparent supervised classification methods that present a model in a tree-like structure, to partition the instances into two distinct classes as homogenous as possible [1]. Classification of the instances starts from the root node and is based on the feature values threshold. At each tree node, the best split is chosen from a set of possible splits to improve on the information gain. The internal nodes (non-final nodes) represent the test node, and the leaf node (end node) represents the decision node that classifiers the instances into one of the two groups. C4.5 and C5.0 are extensions of the ID3 tree method, and C5.0 is an improvement of the C4.5, which addresses the problems of memory, speed and efficiency [3]. Two popular methods used to measure the impurity of a set of candidate splits are:

$$Gini(t) = 1 - \sum_{i=1}^{C} (w_i(t)^2) \qquad \text{and} \qquad Entropy(t) = -\sum_{i=1}^{C} w_i(t). \log_2 w_i(t), \tag{3}$$

where $w_i(t)$ is the proportion of instances belonging to class i at node t.

2.4 Random forest

Random forest (RF) is an extension of DT's proposed by Breiman [4]. Similar to bootstrap aggregation, the RF procedure uses different bootstrap sampling from the original data to build a DT. At each node, a subset of features is randomly selected and the best among all is used to split the node. The final classifier is a collection of weak classifiers aggregated by majority votes (Table 1). The procedure may seem to be computationally intensive, but it is user-friendly because its input involves only two parameters (*ntree* and *mtry*), denoted as N and k in Table 1. Also, RF is robust against over-fitting and several studies have shown effective classification performance with the algorithm.

2.5 Support vector machine

The support vector machine (SVM) is a popular supervised ML algorithm and has been successfully used in pattern recognition with high classification performance results. The

features are mapped into high dimensional feature space the decision boundary (hyperplane) between the classes is constructed based on some selected training data points (support vectors). To minimize errors in classification, the margin separating the classes is maximized. SVM supports both linearly and non-linearly separable classification problems. The SVM classifier satisfies the following condition:

$$\begin{aligned} w, x_i + b &\geq 1 \quad \text{if} \quad y_i = 1, \\ w, x_i + b &\leq -1 \quad \text{if} \quad y_i = -1, \end{aligned} \tag{4}$$

which can be combined into

$$y_i(w, x_i + b \geq 1)$$

Finding the optimal separating hyperplane leads to solving a quadratic programming problem using the Lagrangian multiplier method. The detail of the solution is discussed by Gunn et al. [5] and Vapnik [6]. The final classifier is represented as:

$$h(x) = sign(\sum_{i=1}^{d} \alpha_i y_i K(x, x_i) + b),$$

where α_i represents the Lagrangian multipliers, which are non-zeros for support vectors and $K(.,.)$ is a kernel function.

2.6 Neural network

An artificial neural network (ANN) was included in this study as it has achieved success in pattern classification and has provided the state-of-the-art in most practical settings include image classification, speech recognition etc. [7]. Appropriate application of the ANN often depends on too many possible predictor features. Also, the continuous input features that are of a wide range should be normalised, and influential outliers must be controlled as part of the pre-processing requirements. Although the ANN-based approach has many advantages, one major challenge is the interpretation of the model architecture, especially when many weighted connections are involved [8]. Alternatively, there is a provision to quantitatively describe the ANN model by extracting the variable importance. In this study, we employed a single layer network based on a feed-forward neural network. For a single perceptron (binary class), the logistic or sigmoidal activation function is denoted by

$$Z(\alpha) = \frac{1}{1 + \exp(-\alpha)} \in (-1, 1), \tag{5}$$

where α denotes the neuron input to the activation function, and is a linear combination of the input vectors (x_i) and feature weights (w_i).

Table 1: Pseudocode of the random forest algorithm

Input: S = CRC training dataset, N = no of trees, k = no of features to use at each of the tree
Output: RF tree

1	for $t = 1 : N$	
2	begin	
3	$S_t \leftarrow$	Randomly sample from S with replacement
4	$R_t \leftarrow$	Build a random forest classifier R_t using S_t. For each node n of the tree, recursively repeat the following steps until the specified stopping criterion is reached
5	begin	
6	$k < m \leftarrow$	Randomly select k features that maximize the information gain using equation 3 from the set of m possible candidate features
7	$n \leftarrow$	calculate the node n and the daughter nodes using the best split among k selected features
8	end	
9	Return $R_t = (R_1, R_2, \ldots, R_N)$	
10	for i^{th} new case do	
11	$y_i(-1, +1) \leftarrow$	predict the outcome of the ith new case using rules of R_t
12	$v \leftarrow$	calculate the votes v for each class as predicted in line 11, class with the
13		majority votes is the wining class
14	end	
15	end	
16	Return RF	

Table 2: Hyperparameter values used for model development

	**Recurrence**	**Survival**
LR	Default	Default
NB	fL = 0.5, usekernel = FALSE and adjust = 0.5	fL = 0, usekernel = FALSE and adjust = 0
C5.0	trials = 20, model = tree and winnow = FALSE	trials = 10, model = tree and winnow = FALSE
RF	mtry = 2	mtry = 2
SVM	C = 1	C = 1.65
ANN	size = 6 and decay = 0.1	size = 8 and decay = 0.5

Table 3: The accuracy of each model across the real and simulated data for recurrence and survival studies

Recurrence	WDGMC (N=697)	Sim_Data (N=697)	Sim_Data (N=3485)	Sim_Data (N=6970)
LR	80.1	87.4	84.2	85.0
NB	79.0	85.9	82.7	84.0
C5.0	79.0	84.3	85.0	84.5
RF	80.2	84.2	83.7	84.9
SVM	78.3	87.0	84.4	86.0
ANN	81.0	86.7	86.0	85.0
Survival	WDGMC (N=680)	Sim_Data (N=680)	Sim_Data (N=3400)	Sim_Data (N=6800)
LR	77.0	83.2	82.4	83.0
NB	76.0	83.2	82.2	83.0
C5.0	75.5	80.2	82.2	82.6
RF	76.0	80.2	81.0	82.1
SVM	75.0	82.5	82.3	82.9
ANN	77.0	83.4	82.2	83.1

Table 4: Distribution of patient characteristics across the real and simulated data for all the selected features used in recurrence and survival data

**Variables**	**WDGMC Recurrence 697**	**Sim_Data 697**	**Sim_Data 3485**	**Sim_Data 6970**
**Age at diagnosis**	57.0 (13.0)	57.0 (14.1)	56.4 (13.3)	56.4 (13.4)
**Procedure**				
No	287	256	1417	2862
Yes	410	441	2068	4108
**CRC prior to recruit**				
No	585	587	2922	5835
Yes	112	110	563	1135
**Prior colonoscopy**				
No	246	223	1267	2364
Yes	451	474	2218	4606
**Radiological stage**				
Unable to stage	80	87	395	814
Stage I and II	157	168	756	1538
Stage III	240	232	1227	2452
Stage IV	220	210	1107	2166
**Chemotherapy**				
Yes	246	256	1240	2349
No	451	441	2245	4621
**Previous CRC treatment**				
Surgical	68	64	320	666
Non-surgical	629	633	3165	6304
**Hospital**				
Private	248	247	1217	2467
Public	449	450	2 268	4503
**Treatment decision**				
Chemotherapy	214	234	1020	2126
No chemotherapy	483	463	2465	4844
**Outcome**				
Recur	264	282	1406	2640
Nonrecur	433	415	2079	4330
**Variable**	**WDGMC Survival**	**Sim_Data 680**	**Sim_Data 3400**	**Sim_Data 6800**
**Histology**				
Adenocarcinoma	417	425	2032	4191
Others	263	255	1368	2609
**CRC complications**				
No	375	383	1926	3699
Yes	305	297	1474	3101
**Procedure**				
No	279	287	1387	2777
Yes	401	410	2013	4023
**Hospital**				
Private	243	239	1210	2417
Public	437	441	2 190	4383
**Radiological stage**				
Unable to stage	78	80	414	790
Stage I and II	155	146	776	1528
Stage III	235	229	1175	2339
Stage IV	212	225	1035	2143
**Language**				
English	238	231	1197	2427
Native African Lang.	316	313	1540	3145
Others	126	136	663	1228
**Race**				
White	246	262	1243	2447
Black	356	334	1687	3412
Others	95	84	470	941
**Recurrence status**				
nonrecur	432	416	2152	4388
recur	248	264	1248	2412
**Survival**				
Alive	281	398	1994	3991
dead	399	282	1406	2809

References

[1] Gareth James, Daniela Witten, Trevor Hastie, and Robert Tibshirani. *An introduction to statistical learning*, volume 112. Springer, 2013.

[2] Irina Rish et al. An empirical study of the naive bayes classifier. In *IJCAI 2001 workshop on empirical methods in artificial intelligence*, volume 3, pages 41–46, 2001.

[3] Mevlut Ture, Fusun Tokatli, and Imran Kurt Omurlu. The comparisons of prognostic indexes using data mining techniques and cox regression analysis in the breast cancer data. *Expert Systems with Applications*, 36(4):8247–8254, 2009.

[4] Leo Breiman. Random forests. *Machine learning*, 45(1):5–32, 2001.

[5] Steve R Gunn et al. Support vector machines for classification and regression. *ISIS technical report*, 14(1):5–16, 1998.

[6] Vladimir N Vapnik. An overview of statistical learning theory. *IEEE transactions on neural networks*, 10(5):988–999, 1999.

[7] Terrence L Fine. *Feedforward neural network methodology*. Springer Science & Business Media, 2006.

[8] Marcus W Beck. Neuralnettools: Visualization and analysis tools for neural networks. *Journal of statistical software*, 85(11):1, 2018.
